# Analysis of Filler Spread: Facial Retinacula Play a Prominent Role

**DOI:** 10.1097/DSS.0000000000005088

**Published:** 2026-04-10

**Authors:** Leonie Schelke, Steve Harris, Jair M. Cerón Bohórquez, Nicola Lowrey, Hugues Cartier, Ximena Wortsman, Sebastian Cotofana, Peter Velthuis

**Affiliations:** * Department of Dermatology, Erasmus University Medical Center, Rotterdam, the Netherlands;; † Harris Clinic, London, United Kingdom;; ‡ Medical Contour, Hamburg, Germany;; § N2 Aaesthetics, Los Angeles, California;; ‖ Clinic of Dermatology, Saint-Jean, Arras, France;; ¶ Department of Dermatology, Universidad de Chile, Santiago, Chile;; # Department of Plastic Surgery, Vanderbilt University Medical Center, Nashville, Tennessee

## Abstract

**BACKGROUND:**

Previous ultrasound studies show that unanticipated filler spread can occur during injections.

**OBJECTIVE:**

This study investigates filler spread in common facial locations to determine its predictability based on specific anatomical layers. In particular, the authors focused on the direction of filler spread based on the patterns formed by the retinacula superficialis (RS) and retinacula profundus (RP).

**METHODS:**

In this observational study, the authors analyzed 440 filler treatments performed on 107 patients by a single injector (primary group) and 22 treatments by 4 additional injectors (reference group). Two filler types were evaluated: (1) 2 volumizing hyaluronic acid fillers and (2) 3 biostimulating fillers. The authors also assessed the feasibility of filler testing at the zygomatic arch.

**RESULTS:**

Distinct patterns of filler spread varied by injection site, consistent across both groups. The directional spread was predictable and influenced by the fibrous structures of the RS and RP. In addition, the lengths of the injected bolus varied significantly between the different fillers.

**CONCLUSION:**

In uncompromised tissue, filler spread is confined to the injected tissue layer, but follows directions dictated by the RS and RP fibers. While the overall direction of spread is unaffected by filler rheology, the injected length is influenced by the type of filler used.

## Abstract 2

Supplemental Digital Content is Available in the Text.

Volumizing fillers are widely used in aesthetic practice for augmentation in the supra-periosteal plane and deep fat layers. Traditionally, these fillers were believed to create localized boluses (aliquots) at the injection site. However, ultrasound (US) studies indicate that significant shifts in filler position can occur both during and after injection.^1^ For example, when injected on the zygomatic arch, filler can spread into the temple region.^2^ This has raised concerns, often referred to negatively as “migration,” which implies erratic, unpredictable filler behavior.

In their experience, the authors have observed that when filler is injected on the zygoma just a few millimeters closer to the edge of the maxilla, it frequently moves anteriorly into the deep cheek fat pad. This suggests that certain anatomical areas provide a pathway of least resistance that fillers tend to follow during injection. These pathways may be distinct, yet predictable, for different locations.

Beyond anatomical factors, several other variables may influence filler behavior during injection, including rheological properties such as cohesivity, elasticity, and cross-linking.^3–8^ In addition, factors such as filler volume, injection depth, needle or cannula thickness, and the angle and orientation of insertion (bevel up or down) may affect outcomes.^9–11^ Previous studies primarily used in vitro testing or examinations of body donors.

In this study, the authors used real-time ultrasound imaging to investigate filler spread in commonly injected facial locations. Their objective was to determine whether the directional spread of fillers could be predicted when injected in the appropriate anatomical layer. The authors hypothesized that spread follows patterns established by the retinacula superficialis (RS) and retinacula profundus (RP). In addition, based on this principle of predictability, the authors aimed to analyze filler placement and provide insights for optimizing injection techniques to enhance safety and efficacy.

## Methods

### Study Setup

This observational study consisted of 3 distinct parts, all investigating filler behavior in vivo using ultrasound imaging across a diverse patient population.

#### Part 1

In the primary study, 440 facial areas in 107 patients received ultrasound-guided filler injections between August 2022 and August 2024 (primary group). A facial area was defined as a skin–soft tissue compartment that is, commonly treated with dermal fillers and provides clear visualization of filler behavior during injection. For example, the deep zygomatic fat compartment represents a well-demarcated and widely accepted target area for filler placement. Although injections can also be performed into the superficial fatty layer of the zygoma, this layer is typically relatively thin, making assessment less distinct. Therefore, observations are more reliably obtained within the superficial midfacial fat pad. The treated facial areas are summarized in Table 1. All procedures and imaging were performed in a private medical practice by one of the authors (L.S.), who has more than 15 years of experience in filler injections and ultrasound-guided techniques.

**TABLE 1:** Patient data

Patient data	
Mean age	57,4
Gender	Female
Ethnicity	
Skin Type I	31
Skin Type II	61
Skin Type III	15
Earlier filler treatments	
Prior Filler same area	30
Prior Filler different area	37
Filler Naïve	40

Two filler types were used: (1) volumizing hyaluronic acid fillers and (2) biostimulating fillers. The volumizing fillers included 2 formulations: hyaluronic acid with 1,4-butanediol di-ether (HA-BDDE 20 mg/mL) and hyaluronic acid in polyethylene glycol (HA-PEG 28 mg/mL). The biostimulating fillers consisted of 3 different materials, poly-l-lactic acid (PLLA), calcium hydroxylapatite in methyl cellulose (CaHA), and a combination of hyaluronic acid with calcium hydroxylapatite (HA-CaHA). Poly-l-lactic acid was prepared by adding 5 mL of sterile water to the powder in the vial, shaking, then adding another 2 mL of sterile water. Before injection, 2 mL of 1% lidocaine was added. The choice of filler was determined by each patient's specific needs, either volume restoration or skin quality improvement. HA filler was used for volume restoration and injected at the periosteal level. For cases in which skin improvement was the primary objective, a biostimulatory filler was placed in the superficial fat layer. When mild volume loss was present in these cases, the biostimulatory filler was also used to provide additional volume. HA-CaHA hybrids were excluded in the latter cases because they are not approved for deep fat injection.

#### Part 2

For corroboration, and to assess whether filler behavior and spread was consistent across different injectors, between July 2024 and September 2024, 4 additional medical providers—all skilled in facial ultrasound anatomy and ultrasound-guided injection techniques—examined 2 or more additional patients using the same methodology (reference group). This group consisted of 22 treatment areas.

#### Part 3

The third part quantitatively assessed filler spread on the zygomatic arch. The purpose of these measurements were to determine whether this region represents a predictable site for filler behavior in vivo. The zygomatic arch was selected for its reproducible ultrasound imaging due to the horizontal bone and fascia separating the lateral suborbicularis oculi fat and the prezygomatic fat. Injecting on the periosteum results in predictable backflow,^1^ easily captured by ultrasound. For each filler type, lengths of the horizontal spread of standard 0.2-mL injected boluses were measured using a limited subset of 10 measurements. HA-CaHA was omitted from this assessment as it is not intended for deep fatty layers. Statistical analysis was performed using chi-squared and Fisher exact tests to compare differences in the length of spread for the various injected fillers (SPSS 29.0; IBM, Armonk, NY)—with a two-tailed *p*-value of ≤.05 indicating statistical significance.

Consecutive patients seeking filler treatment at the clinic of one of the authors (L.S.) were included in the study. Patients included were either treatment-naïve or presented for repeat treatment in a new facial area or a repeat filler treatment in the same facial area. In the last category, for those who had received filler treatment in the specified locations within the past 18 months or had remnants of previously injected filler detected by ultrasound imaging were excluded. Patients who had undergone facelifts or other facial surgeries that may have influenced the retinacula were excluded as well. No confounders were predefined; therefore, none were included in the statistical analysis. All participants provided written informed consent for the use of their data, including videos and images of the ultrasound examinations.

The study adhered to the principles outlined in The Medical Research Involving Human Subjects Act and the Helsinki Principles of Medical Ethics. Each patient signed an informed consent form before participating in the study.

### Ultrasound Imaging

**Figure 1.**
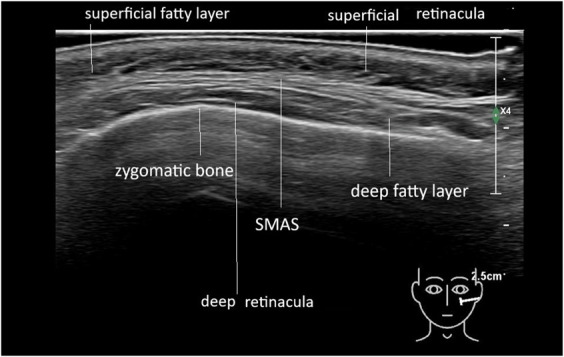
Ultrasound images of normal skin, superficial fat, SMAS and deep fat, the retinacula cutis superficialis and profundus. SMAS, superficial musculo-aponeurotic system.

Ultrasound imaging of the face reveals 3 primary layers between the dermis and bone: superficial fat, deep fat, and fibrous tissue structures that separate them, visualized as hyperechoic lines parallel to the skin surface (Figure 1). These fibrous lines represent the superficial musculo-aponeurotic system (SMAS), with hypo- or anechoic structures indicating fat or muscle fibers between them.

On ultrasound, a fat pad appears as hypo/anechoic pockets separated by hyperechoic lines, presumed to be fibrous tissue. The fibrous structures in the superficial fat pads are termed RS and those in the deep fat pads RP.^12^

**Figure 2.**
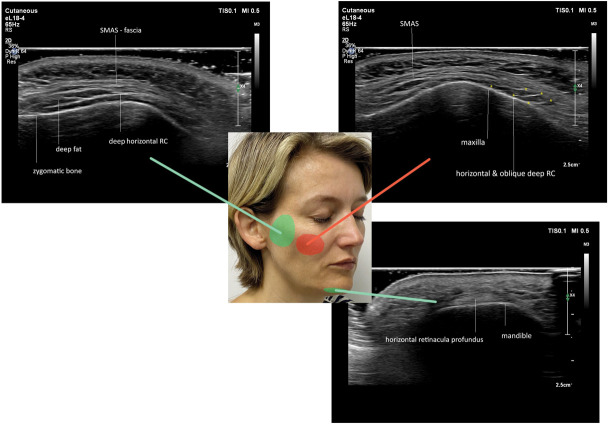
Distribution of hyperechoic lines in the deep fatty areas in the face. Green displays the horizontal pattern (A) and red displays the oblique lines (B). Female patient aged 44 years.

**Figure 3.**
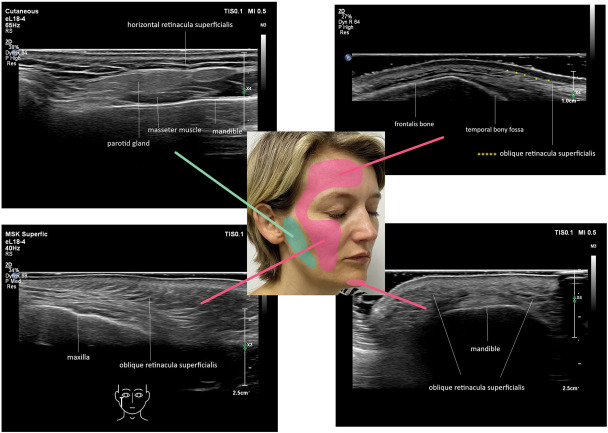
Distribution of hyperechoic lines in the superficial fatty areas of the face. Pink for those with an oblique pattern (A) and green for those with a horizontal pattern (B). Female patient aged 44 year old.

The RP run mainly parallel to the skin surface, except in the maxillary fat pad where they course obliquely, connecting the SMAS to the periosteum or deeper fibrous structures (Figures 2 and 3).

**Figure 4.**
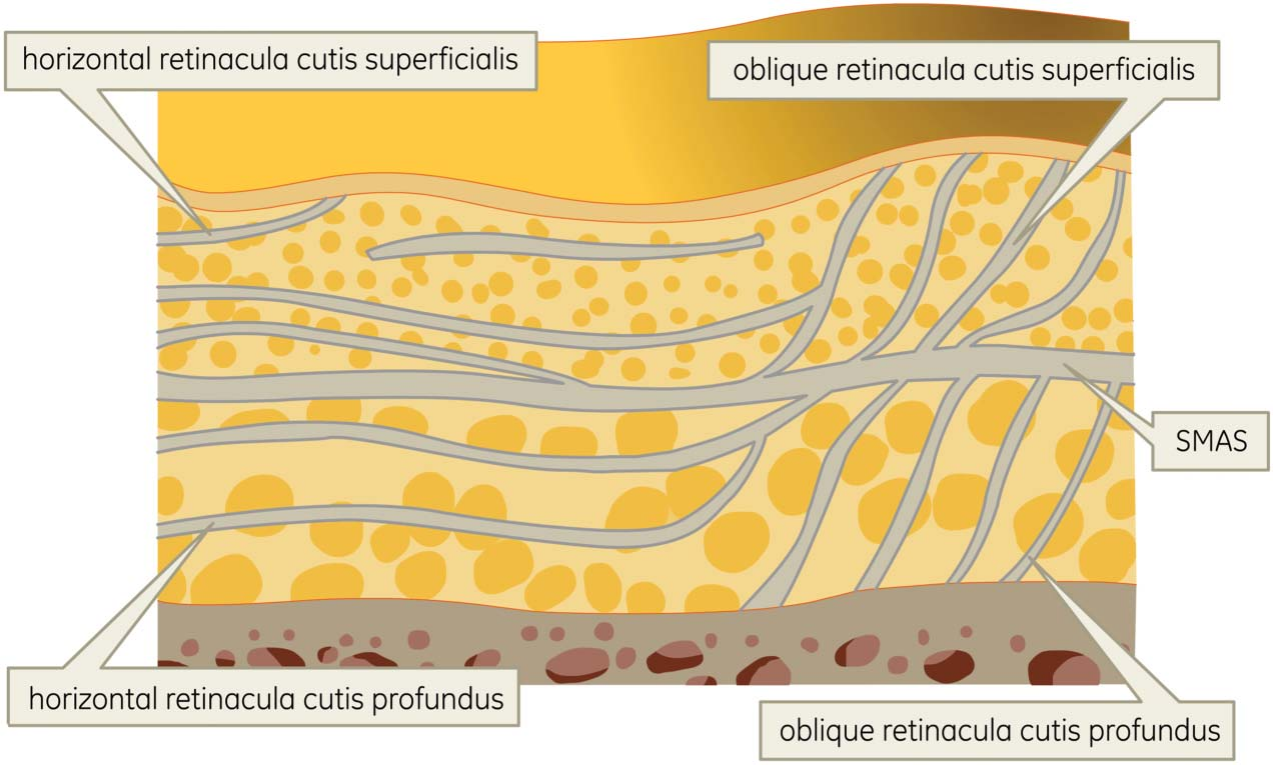
Schematic drawing of the different facial patterns of the retinacula superficialis and profundus. The drawing shows an overall general direction of fibers, not a specific facial area.

By contrast, within the superficial fatty layers of the midface, temples, and forehead, the RS are primarily oblique (Figure 4A), while in the lateral and horizontal jawline, their orientation is predominantly parallel to the skin surface (Figures 3 and 4).

### Ultrasound-Guided Filler Injections

A high-frequency MHz transducer, either an 18-MHz linear probe Philips Affinity 70 (Philips Eindhoven, The Netherlands), or a 20-MHz linear probe GE Venue Go/Venue Fit (GE Healthcare Venue Go, Chalfont St Giles, United Kingdom), was used for filler injections with simultaneous ultrasound visualization (ultrasound-guided). In-plane visualization was primarily used for injections using a 27-G needle or a 25-G cannula with an estimated 15-30 to 120° angle to the skin. Out-of-plane visualization was used for 90° angled injections with a 27-G needle perpendicular to the deep fat layers above the periosteum. When placed in the deep fat on the zygoma, a bolus of 0.2 mL was injected with HA filler. HA-CaHA was not used on the zygoma as that would be an off-label procedure.

In the superficial fatty layers, a retrograde tunnelling technique was applied for HA-BDDE, HA-PEG, and all types of biostimulating filler injections. During ultrasound-guided filler injections, the spread of the filler through the deep and superficial fat was observed. Ultrasound images/videos were stored, and to avoid interpretation bias, further assessed independently by 2 physicians experienced in interpreting ultrasound imaging.

During injection, filler flow was recorded using a probe position that was standardized for each anatomical area. Immediately after injection, filler spread was assessed by sliding the probe in all directions—and rotating it when necessary—to identify the predominant direction of flow, as reported in Table 1. Videos were recorded to capture the tip of the needle or cannula allowing observation of the filler behavior during injection. Any discrepancies in the images led to their exclusion from the study.

## Results

### Sample Description

In the primary study, 440 facial areas (skin–soft tissue compartment commonly treated with dermal fillers, see Table 1) were treated across 107 patients, all of whom were female. Most patients presented for repeat treatment, while 18 patients were treatment-naïve. The mean age of participants was 57.4 years (SD 6.9; range 43–73). Thirty-one patients had skin Type I, 61 had skin Type II, 15 had skin Type III, and none had skin Type IV or V (Table 1).

The second part of the study, the reference study consisted of 22 facial areas across 10 patients (reference group). Results are summarized in Table 1.

In the third part of the study, 40 facial areas of 20 patients from the primary study were analyzed for filler measurements. All were female, with a mean age of 55.7 years (SD 9.7; range 43–84).

### Filler Behavior Observations

**Figure 5.**
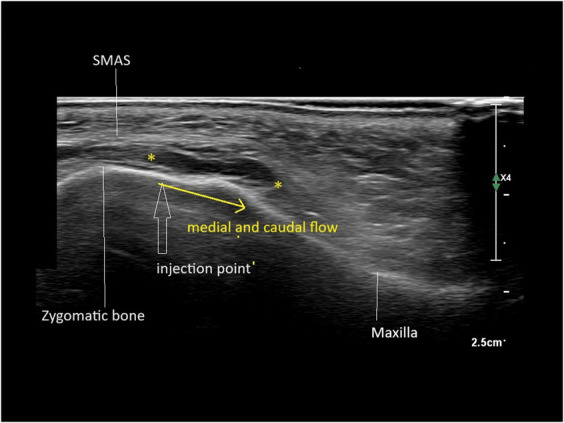
Medial and caudal flow between the oblique deep retinacula cutis. Hypoechoic (black) HA between markers.

The injected HA fillers were intended to form small aliquots. However, when more than a minimal volume (e.g., >0.05 mL) was injected, the filler was observed to spread, resulting in oval-shaped or longitudinal pockets. This spread appeared to be directionally guided by the arrangement of the retinacula in both the superficial and deep fat pads (Figures 4 and 5; **Supplemental Digital Contents 1–3**, **Videos 1–3**, http://links.lww.com/DSS/B853, http://links.lww.com/DSS/B854, and http://links.lww.com/DSS/B855). Importantly, the dispersion patterns were specific to each injection site and consistent across all patients (Table 2 for details). Variables such as the angle of the needle (30, 90, or 120°), the choice of needle versus cannula, and the size of the instrument did not influence the flow pattern of the filler material.

**TABLE 2:** Dispersion patterns of filler spread

Location (US Pattern of Fibrous Lines)	Number of Facial Area's	Filler Type	Needle/Canula/Gauge-Size	Injection Angle in Degrees	Direction of Filler Movement
Main group					
Zygoma deep fat (horizontal RC pattern)	64	HA	Needle 25G	30	Lateral flow
	6	HA	Needle 27G	90	Lateral flow
	6	HA	Needle 25G	−30	Lateral flow
	12	HA-PEG	Needle 25G	30	Lateral flow
	48	HA-CaHA	Needle 25G	30	Lateral flow
	26	PLLA	Needle 25G	30	Lateral flow
Maxilla deep fat pad (oblique RC pattern)	20	HA-PEG	Needle 25G	30	Medial and caudal flow
	20	HA	Needle 25G	30	Medial and caudal flow
Pyriform space (no RC)	10	HA	Needle 27G	30	Cranial flow
Mental protuberance (oblique RC pattern)	20	HA	Needle 25G	30	Sideways and more superficial
Mandible deep fat (horizontal RC pattern)	10	HA	Needle 25G	30	Lateral flow
Jawline superficial fat (horizontal RC pattern)	64	HA-CaHA	Canula 25G	90	Lateral flow
	10	PLLA	Canula 25G	90	Lateral flow
	10	HA	Canula 25G	90	Lateral flow
	10	CaHA	Canula 25G	90	Lateral flow
Preauricular (horizontal RC pattern)	10	PLLA	Canula 25G	90	Caudal flow
	10	HA	Canula 25G	90	Caudal flow
	10	HA-CaHA	Canula 25G	90	Caudal flow
Forehead preseptal fat (horizontal RC)	10	HA	Needle 25G	15	Lateral flow
Interfascial plane temple (oblique RC)	10	HA	Needle 27G	90	Caudal flow
Midface superficial fat (oblique RC)	10	HA	Canula 25G	90	Caudal flow
	26	PLLA	Canula 25G	90	Cauda flow
	48	CaHA	Canula 25G	90	Caudal flow
Reference group					
Zygoma deep fat	6	HA	Canula 22 & 23G	NS	Lateral flow
			Needle 25G	NS	Lateral flow
Maxilla deep fat	10	HA	canula 22&25G	NS	Medial and caudal flow
			Needle 25 & 27G	NS	Medial and caudal flow
Medio jugal (tear trough) deep fat	1	HA	Canula 22G	NS	Caudal flow
Mandible deep fat	2	HA	Needle 27 g/canula 25G	NS	Lateral flow
Jawline deep fat	1	HA	Canula 25G	NS	Lateral flow
Interfascial plane temple	2	HA	Canula 23G	NS	Caudal flow

CaHA, calcium hydroxylapatite; HA-CaHA, Hyaluronic Acid Calcium Hydroxyl–Apatite combination; HA, Hyaluronic Acid cross-linked with BDDE (1,4-Butanediol Di-Ether); HA-PEG, Hyaluronic Acid crosslinked with PEG (Poly-Ethylene Glycol); NS, Not-Studied; PLLA, Poly-l-Lactic Acid; RC, retinaculum cutis; US, ultrasound.

Table 2 summarizes locations and details of filler injection techniques and filler behavior in the primary study (*N* = 440) and reference group (*N* = 22).

**Figure 6.**
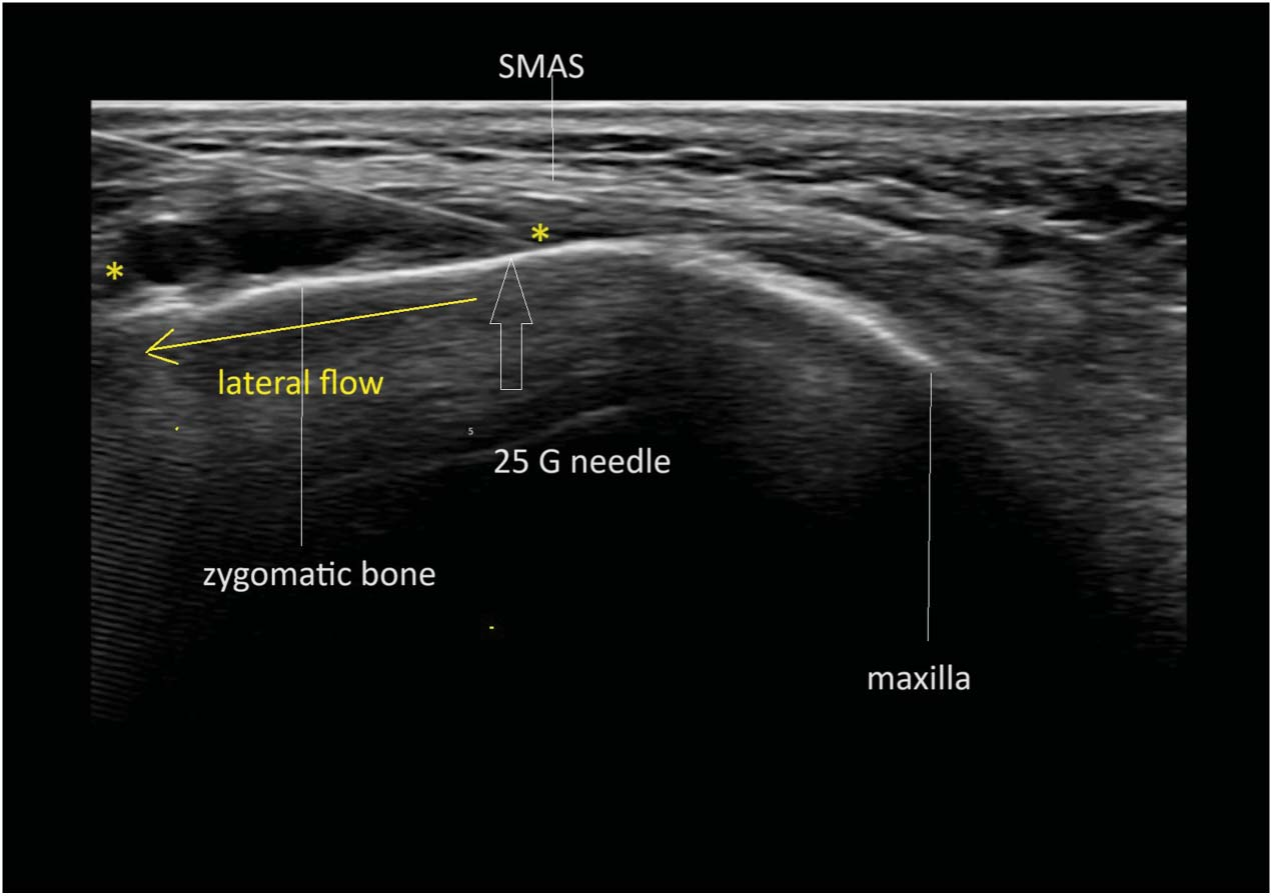
Lateral flow between the horizontal deep retinacula cutis. Hypoechoic (black) HA between markers.

The movement patterns of biostimulatory fillers did not differ from those of HA fillers. When injected as a bolus in the deep fat, the filler lifted the fascia or deep retinacula above it to a certain extent. After maximal lifting, depending on the specific pattern of the retinacula in that area, the filler was observed to flow either backward or forward. (Figure 6).

**Figure 7.**
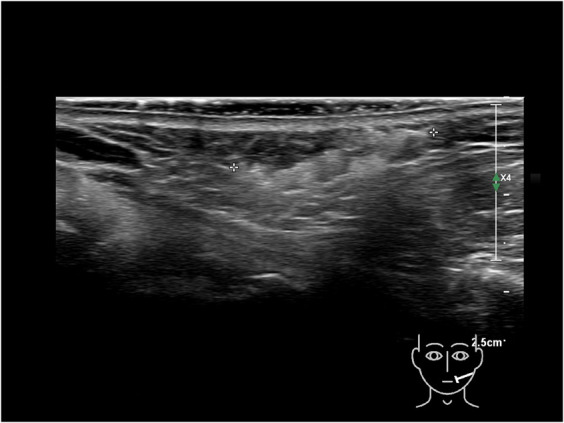
Caudal flow between the oblique superficial retinacula cutis. Hyperechoic (white) HA-CaHA between markers.

Using a linear retrograde technique in the superficial fat of the preauricular area, where the retinacula run parallel to the skin, all filler substances (HA, CaHA, HA-CaHA, and PLLA) appeared to fill and enlarge the injection tunnel formed by the cannula. Further spread of the material was restricted by the RS and SMAS (Figure 7; **Supplemental Digital Content 4**, **Video 4**, http://links.lww.com/DSS/B856).

**Figure 8.**
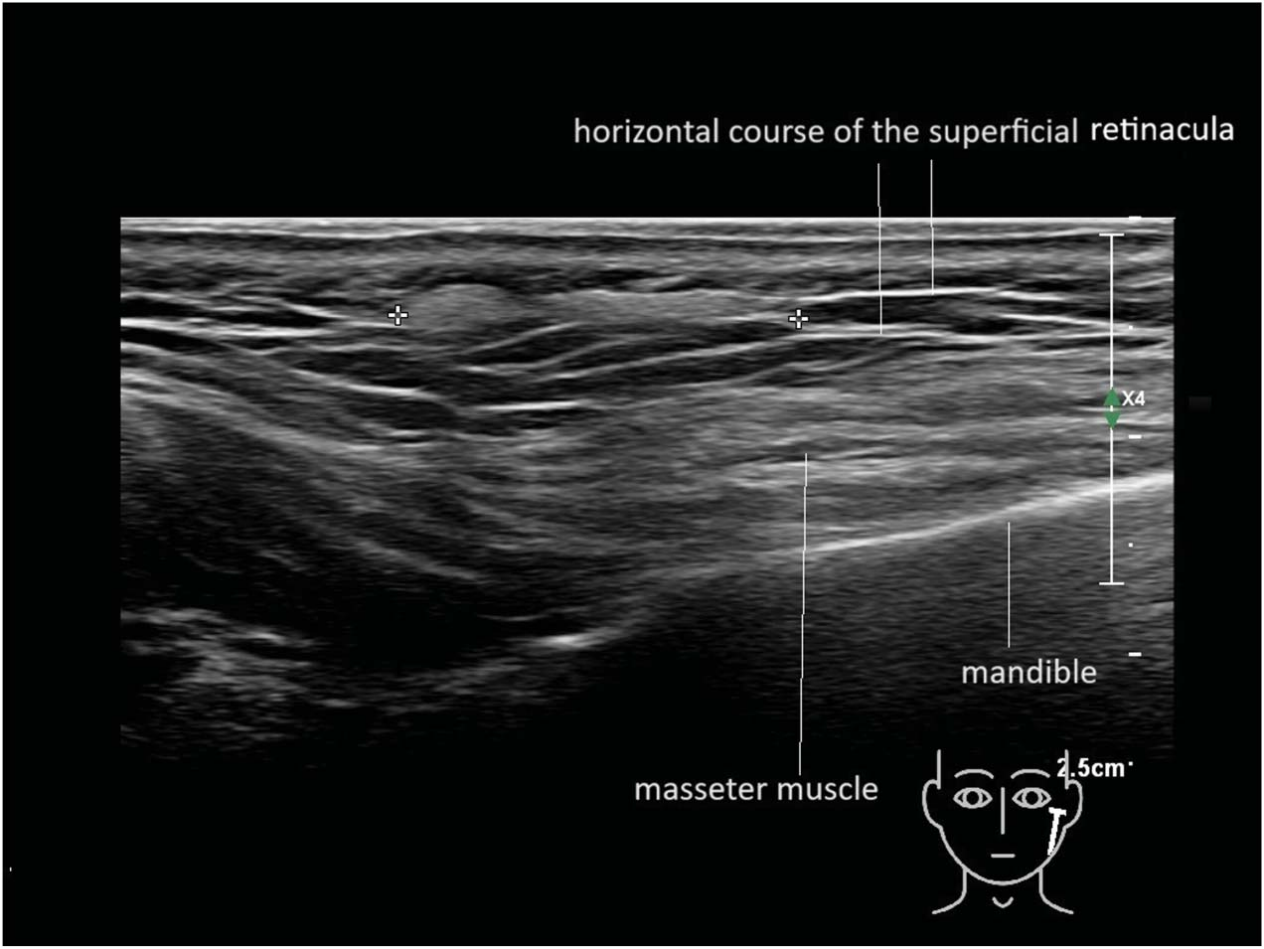
Lateral flow between the horizontal superficial retinacula cutis. Hyperechoic (white) CaHA between markers.

By contrast, when fillers were injected into the superficial fat of the midface, just beneath the dermis where the RS sheets run obliquely, some material descended from the dermis to the SMAS, following the oblique orientation of the RS. This specific movement was guided by the RS (Figure 8; **Supplemental Digital Content 5**, **Video 5**, http://links.lww.com/DSS/B878). Notably, this phenomenon of filler spread in the superficial fatty layer was not dependent on the injected volume.

### Quantitative Analysis

For the quantitative study, a power calculation was performed based on the preobserved substantial difference between groups, with an effect size of 1.5, an alpha error probability of 0.05, and a power of 0.90. For statistical analysis, a one-way ANOVA test was performed in SPSS 30.0, with *p* < .05 considered significant (Figure 8).

The measured lengths of a 0.2-mL injected filler bolus were as follows:

HA-BDDE cross-linked (20 mg/mL): 2.05 cm (SD ± 0.25 cm); HA-PEG cross-linked (28 mg/mL): 1.80 cm (SD ± 0.11 cm)

CaHA: 1.13 cm (SD ± 0.24 cm).

PLLA: 1.37 cm (SD ± 0.21 cm).

**Figure 9.**
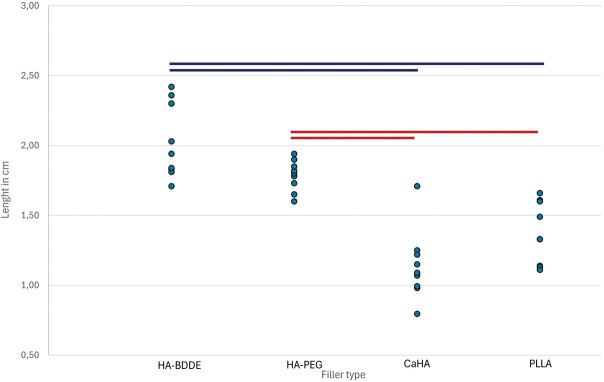
Length (cm) of 0.2-mL injected filler boluses on the periosteum of the zygoma. Blue and red lines connect groups with significant differences (*p* < .001 in each).

The HA-BDDE cross-linked and HA-PEG cross-linked fillers spread significantly farther than both CaHA and PLLA. However, there was no significant difference between the 2 HA cross-linked fillers (HA-BDDE vs HA-PEG, *p* = .053) or between the biostimulatory fillers (CaHA vs PLLA, *p* = .066) (Figure 9).

## Discussion

This study examined filler treatments under ultrasound guidance in 440 facial areas (primary group), with a reference group of 22 facial areas injected by 5 experienced providers (reference group). Care was taken to ensure that injections were performed either into the superficial or deep fatty layers, avoiding placement between the parallel hyperechoic lines that indicate the SMAS or fascia. Remarkably, the pattern of filler movement demonstrated consistency across locations, suggesting that tissue resistance and surrounding retinacular structures are key determinants of filler behavior, which appear to be largely identical among individuals.

The hyperechoic lines in the fatty layers, representing fibrous tissue, create a three-dimensional structure known as the *retinacula superficialis* and *retinacula profundus*. While ultrasound imaging provides a 2D representation, a hyperechoic line may depict a cord, a small membrane, or a more extensive sheet. These structures connect to the SMAS, with the RP anchoring on the deep side to the periosteum and the RS on the superficial side to the dermis.^12–15^ This intricate architecture is significant because most facial muscles are closely linked to the SMAS, allowing for the transmission of mimetic movements to the skin through the RS. In the superficial fatty layers, the retinacula compartmentalize the fat into lobules. In areas devoid of fat, the retinacula are densely packed, forming ligament-like structures.^16^

The findings of this study indicate that the spread of filler follows distinct directional pathways—namely lateral, medial, cranial, or caudal—yet consistently remains within the same plane, either superficial or deep to the SMAS. For example, during the injection of a filler bolus on the periosteum of the zygomatic arch, when pressure from the surrounding tissue builds up and surpasses a certain threshold, the filler disperses laterally. When injection volumes in this specific area exceed 0.2 to 0.3 mL, the filler continues to spread from the zygoma into the caudal temple.^1^ In the midface, injections at the border of the zygoma and the maxilla result in medial and caudal spread.

In the deep fat pad of the chin, the *retinacula profundus* exhibit different anatomical characteristics, with fat and fibrous tissue more intermixed (resulting in fibro-fatty tissue characteristic of that area). The spread of filler here tends to be lateral and superficial, likely traversing the spaces between fibers of the mentalis muscle.

In the superficial fat pads, the retinacula may be oriented parallel or oblique to the skin surface. When retinacula are parallel, filler distribution flows along and between these fibrous septa. When the *retinacula superficialis* run obliquely, the filler follows the retinacular pattern downward. In their current understanding, filler is injected into either the deep or superficial fat; however, these fatty layers are densely packed with fibrous sheets. Instead of simply shaping a fatty compartment, these fibrous structures guide filler spread in specific directions. Further research is required to understand the implications for clinical outcomes.

Future research should explore the anatomical mapping of the retinacula. In addition, longitudinal studies examining how repeated treatments influence tissue structures and filler behavior over time could provide valuable insights into long-term aesthetic outcomes.

The difference in the lengths of the 2 HA cross-linked fillers (*p* = .05) is on the verge of statistical significance. A larger sample might reveal a significant difference. Injecting HA-PEG was found to require more force than injecting HA-BDDE.

Investigating the effects of different filler characteristics and rheological properties on spread dynamics is also essential.^11–13,17–21^ The behavior of fillers over time is a crucial consideration for ongoing treatment strategies. As fillers gradually degrade, understanding predictable spread patterns can enhance the development of effective maintenance protocols that account for the longevity of the desired effects. This could also facilitate more consistent aesthetic outcomes across multiple treatment sessions.

There are several limitations to this investigation. The convenience sample may have introduced selection bias. Prior filler treatment could have altered tissue characteristics and influenced filler spread. However, their unpublished observations suggest that while prior treatments may affect the speed and volume of diffusion, the direction and pattern remain largely consistent. Although all fillers followed similar directional pathways at each facial location, the extent of movement appeared to depend on the type of filler used. While measuring the length of spread can provide insights into filler characteristics, it is important to note that the SMAS and surrounding fascia are three-dimensional structures. Therefore, more accurate assessments than length alone—such as volume measurements, monitoring filler spread over time, and evaluating rheological properties—are necessary. In addition, anatomical variations, including differences in retinacular structure, were not considered in this study.^22^ Only women were treated in this study. Research has indicated disparities in retinacular density both among individuals and between genders.^23^ Developing a detailed 3D map of retinacular patterns would be beneficial for future studies.

Age-related factors may also influence filler diffusion. Further research addressing possible age-, sex-, and ethnicity-related variables is essential to enhance their understanding of filler behavior and to optimize injection techniques.

More emphasis should be placed on assessing individual anatomical variations when determining injection strategies. Recognizing unique retinacular patterns and tissue characteristics in each patient could further enhance treatment outcomes and patient satisfaction. For example, the effectiveness of different filler types may vary according to the anatomical context and individual patient characteristics.

## Conclusion

This study demonstrates that filler spread during ultrasound-guided injections is predictable and closely follows anatomical structures, specifically the patterns of the retinacula superficialis and retinacula profundus, which seem to be major determinants of directional filler spread both during and after injection. For each facial location examined, fillers exhibited consistent and predictable patterns of dispersion. In uncompromised (healthy, undisturbed) tissue, the spread remains confined to the injected tissue layer and is not influenced by filler rheology.
